# Virtual Connections: Improving Global Neurosurgery Through Immersive Technologies

**DOI:** 10.3389/fsurg.2021.629963

**Published:** 2021-02-19

**Authors:** George Higginbotham

**Affiliations:** North Bristol National Health Service (NHS) Trust, Bristol, United Kingdom

**Keywords:** virtual reality, augmented reality, global neurosurgery, COVID-19, immersive technologies

## Abstract

The field of neurosurgery has always been propelled by the adoption of novel technologies to improve practice. Although advancements have occurred in the diagnosis, treatment, and long-term outcomes of patients, these have not translated to global patient benefit. Up to five million people each year do not have access to safe and affordable neurosurgical interventions, and those in low- and middle-income countries (LMICs) are disproportionately affected. Current approaches to increase neurosurgical capacity are unlikely to meet the UN Sustainable Development Goals target by 2030, and many of the most successful programs have been disrupted by the travel restrictions of the COVID-19 pandemic. There is therefore a pressing need for creative virtual solutions. An area of growing relevance is the use of immersive technologies: virtual reality (VR) and augmented reality (AR). AR allows additional information to be superimposed onto the surgeon's visual field, thus enhancing intra-operative visualization. This can be used for remote tele-proctoring, whereby an experienced surgeon can virtually assist with a procedure regardless of geographical location. Expert guidance can therefore be given to both neurosurgical trainees and non-neurosurgical practitioners, further facilitating the growing practice of neurosurgical task-shifting in LMICs. VR simulation is another useful tool in remote neurosurgical training, with the potential to reduce the learning curve of complex procedures whilst conserving supplies in low-resource settings. The adoption of immersive technologies into practice is therefore a promising approach for achieving global neurosurgical equity, whilst adapting to the long-term disruptions of the pandemic.

## Introduction

The twenty-first century has seen exponential growth in the development and adoption of novel technologies across many aspects of society, and medicine and surgery is no exception (1). Technology occupies a growing role in healthcare delivery, with particular impact on surgical and perioperative care (2). Neurosurgery in particular has been a speciality driven by technological innovation; advancing the diagnosis, treatment, and long-term outcomes of patients (1).

However, these advancements have not benefitted patients equally worldwide. There is growing disparity in the provision of surgical care globally, with significant inequalities seen in neurosurgical care (3). Up to five million people each year do not have access to safe and affordable neurosurgical interventions, the majority of such patients living in low and middle-income countries (LMICs) (4). This disparity is responsible for significant morbidity and mortality worldwide (5), rendering the search for creative solutions of utmost importance.

In 2015, the United Nations General Assembly adopted a 17-goal action plan to transform the world by 2030. These Sustainable Development Goals set out key aspirations for global healthcare, a crucial component being the provision of surgical care (6). In the same year, the Lancet Commission on Global Surgery was formed to help elucidate the true burden of unmet need in surgical care, identifying important areas for improvement (7). With 10 years remaining until the 2030 deadline, we need systemic change in healthcare systems on an international scale to ensure these vital goals are met.

The development and application of novel technologies will play a vital role in ensuring equity in global neurosurgery, whilst fostering advancements in future neurosurgical care. A particularly promising field is immersive technology, demonstrating potential applications in pre-operative planning, intraoperative guidance, and neurosurgical training. Adoption of these technologies is particularly relevant in the context of the COVID-19 pandemic, as we seek virtual solutions to disruptions in surgical care and education.

## Current Global Neurosurgical Landscape

In comparison to other surgical specialities, the burden of unmet need in neurosurgery is particularly great. Every year, an estimated 22.6 million patients suffer from neurological disorders or injuries that warrant neurosurgical input. Of these, 13.8 million require surgery, but an estimated 5 million remain untreated each year (4). Episodic service missions from developed countries cannot fill these gaps sustainably (8), and instead international support must center around training and education (5). It is well-accepted that the most successful global health interventions must be designed with the recipient population involved at all stages of development (9), with the resulting approach focusing on the empowerment of local healthcare users and providers (5).

In many LMICs, the neurosurgical workforce capacity is only 1–10% of the minimum expected neurosurgeon ratio per population (4), with over 23,000 more neurosurgeons needed to address this deficit by 2030 (10). Multiple approaches have been considered to address this gap in essential neurosurgical provision; for example, through visiting fellowships in which local surgeons learn from centers in higher-income countries (HICs). However, these fellowships can result in “brain drain” in LMICs, as the neurosurgical workforce is further depleted whilst surgeons train overseas, and may indeed stay for extended periods (11). A solution to this issue is the development of neurosurgical training programs based in, or near the country of need.

One successful example is the CURE program in Sub-Saharan Africa, where surgeons in Uganda and the surrounding countries were trained in an alternative, low-cost procedure to treat hydrocephalus, in response to high disease incidence and limited supply of shunts. The programme has now expanded to 15 global sites (12). A more comprehensive approach to training is through a twinning paradigm, such as the Swedish African Neurosurgical Collaboration. This is a multi-stage model featuring utilization of local resources, targeted donations of equipment, and reciprocal clinical visiting partnerships (8).

Despite these multifaceted approaches to increase neurosurgical capacity in LMICs, the deficit remains. Whilst the global neurosurgical workforce is increasing, 58% of LMICs will not meet the minimum target for neurosurgical workforce density by 2030 (13). An alternative strategy aims to address the gap in care provision without requiring additional neurosurgeons. In task shifting and task sharing, aspects of neurosurgical care are delegated to non-neurosurgeons (i.e., general surgeons, general practitioners, and non-physician clinicians) with differing levels of supervision and autonomy (14). These approaches remain controversial. Whilst task sharing may be the most time-effective way of addressing the lack of access to essential neurosurgical care, it suffers from a lack of longevity, fails to ensure that adequate structures are in place to carry out more complex neurosurgical procedures, and does not address deficits in training numbers.

## Role of Immersive Technologies in Neurosurgery

The term “immersive technologies” primarily refers to the concepts of virtual reality (VR) and augmented reality (AR). In VR, a computer-generated image or environment is simulated, and can be interacted with by the user. AR combines both virtual and real objects in a single view, thus producing a semi-immersive environment (15). Within surgery, immersive technologies can therefore be used to create 3D simulations of anatomical structures, which can then be superimposed onto views of a patient's real anatomy.

Of all the surgical specialities, neurosurgery may benefit most from technologies which improve the visualization of structures. The closed nature of the cranium and spine, and complex micro-anatomy within, poses a natural limitation on operations that can be performed without invasive methods of exposure. Neurosurgical care has been revolutionized by advances in imaging technology, with modern neurosurgery becoming heavily reliant on imaging for surgical success. Routine pre-operative and intra-operative use of computed tomography (CT) and magnetic resonance imaging (MRI) has facilitated the use of less invasive techniques, whilst improving surgical accuracy (1).

These imaging modalities are used in stereotactic surgery, where scans are combined with the intraoperative view to allow for accurate neuro-navigation (1). This is a basic form of augmented reality already in common use in neurosurgery, and so the leap to more complex immersive technologies is a natural one, allowing for information from scans to be assimilated more effectively. Traditional neuro-navigation systems utilize a “heads up” approach, with the computed images displayed separately from the intraoperative field (16). The surgeon must repeatedly switch their view from the computer screen to the operative field, disrupting surgical workflow (15).

Integration of the navigation system with the operative field would remove the need to switch views and would therefore provide a more intuitive experience. The ability to view 3D images superimposed onto the operative field has been shown to provide enhanced visualization of neurovascular structures, and improved intra-operative lesion location in neuro-oncological surgery (17).

Portable forms of immersive technologies, such as smart glasses, could vastly improve the usability of existing navigation systems, extending their use beyond the theater setting for use in pre-operative planning and surgical training. Rather than an AR overlay, a VR model of the patient's individualized anatomy could be created, allowing the surgeon to perform an accurate trial of the surgery with no associated patient risk. The availability of more realistic operative simulations has a wider applications neurosurgical training, allowing for greater understanding of complex anatomy and honing of visuospatial skills (1, 18, 19), thus reducing the learning curve for complex procedures (19).

## Immersive Technologies in LMICs

The Lancet Commission recognized that novel technologies are key factors in enabling the scaling up and strengthening of surgical care worldwide (9). The adoption of novel technologies will allow for reduced costs, optimize the use of resources, and improve the delivery of care and training (7). Specifically, simulation-based approaches have been identified as a useful tool in surgical training in LMICs, ensuring that critical steps of a high-risk procedure can be practiced in a low-risk environment, preserving patient safety and scarce hospital supplies (7). The NIHR Global Health Research Group in Surgical Technologies (GHRG-ST) are currently trialing surgical simulation training that incorporates immersive technologies in Sierra Leone with promising results (20, 21).

A growing practice in surgical training globally is the intraoperative use of tele-mentoring and tele-proctoring. Rural surgical trainees have highlighted the relevance of surgical tele-mentoring in the acquisition of new surgical techniques and skillsets (22). Tele-proctoring uses internet connectivity to provide an audio and visual connection between surgeons in different geographic locations (23). In this manner, the mentoring surgeon can provide the real-time guidance and assistance typical of traditional surgical mentoring without being physically present. This field could be dramatically enhanced through the application of immersive technologies, allowing the mentoring surgeon to better demonstrate procedural steps through a combination of hand gestures, annotations, and imaging overlaid on the operative field (24). This has been demonstrated in complex surgical procedures, including those performed by local surgeons in LMICs with assistance from mentors in HICs (23–25). Of note is the application in neurosurgical procedures such as endoscopic third ventriculostomy, where guidance could be given with high levels of precision (25).

Although the financial cost of procuring equipment is a common barrier to the adoption of novel technologies in LMICs (9), many of the immersive technology innovations have been designed to make use of existing equipment and software. The use of comparatively low-cost mobile devices is a growing phenomenon in LMICs healthcare systems, with supporting evidence of their efficacy (26). For example, VIPAR is an iPad-based platform that allows mentoring surgeons to project their hands into the display of the mentee surgeon (27). Furthermore, many of the AR and VR headsets can be used *via* a smartphone (20, 21), with further scope to utilize low-cost headsets such as Google Cardboard (16).

This technology may also be used as an adjunct to task shifting and task sharing in neurosurgery, adding an additional level of specialist support to non-neurosurgical practitioners. Such applications have been considered for rural hospitals or military medical centers, where general surgeons may be tasked with performing complex sub-speciality procedures, including craniotomies (28).

These solutions utilize the increasing ease with which we can connect with colleagues across the world, traversing national and continental barriers. It is clear that wearable immersive technologies and web-based communication can contribute to a sustainable model of surgical training in resource-poor locations (29).

## Relevance To COVID-19 Pandemic

The COVID-19 pandemic has presented a huge burden to healthcare systems across the globe, particularly in LMICs (30). Beyond the immediate threat of the pandemic, the pre-existing burden of unmet neurosurgical need remains, and has been exacerbated. In neurosurgery, many treatments are time-critical, and any delays to treatment may cause significant morbidity and mortality (31). Additionally, many of the models designed to improve neurosurgical training, such as twinning programmes and exchanges, cannot feasibly take place under international travel restrictions. These limitations on in-person training may remain for some time, risking long-term disruption to surgical care and training; the effects of which will be felt for years to come (32–34).

In response to the pandemic-driven disruption, there has been a surge of innovation in virtual methods of education and collaboration. Worldwide, face-to-face teaching has been replaced by webinars and online courses (31, 33). Proximie is a secure, cloud based AR platform that has been used for surgical education, providing interactive masterclasses and tutorials (16, 35). From classroom to operating room, augmented reality telesurgery has been successfully used to provide expert guidance when operating on COVID-19 positive patients, both limiting the number of professionals in the room and conserving vital supplies of personal protective equipment (36). These virtual solutions are examples of low-cost innovations that can be utilized by LMICs and HICs.

## Conclusion

Neurosurgery has always been a pioneering speciality, harnessing developing technologies to improve patient care. Immersive technologies are one such tool with growing potential to be adopted over the next decade. A focus for improvement is the global discrepancy in neurosurgical care, which leads to significant morbidity and mortality annually, and exacerbates inequalities between HICs and LMICs. The current COVID-19 pandemic has emerged at this intersection between rapidly developing technologies and the lack of equal access worldwide. The restrictions to daily life have challenged traditional models of surgical care and training, and there has been rapid adoption of novel technologies in response. This innovation presents an opportunity to ensure solutions work to improve neurosurgical care in LMICs as well as HICs. Although it is uncertain what challenges neurosurgery may face in the next 10 years, it is clear that a better connected and equal global neurosurgical workforce will put us in the best position to face these challenges.

## Data Availability Statement

The original contributions presented in the study are included in the article/supplementary material, further inquiries can be directed to the corresponding author/s.

## Author Contributions

The author confirms being the sole contributor of this work and has approved it for publication.

## Conflict of Interest

The author declares that the research was conducted in the absence of any commercial or financial relationships that could be construed as a potential conflict of interest.
